# Revolutionizing nematode management to achieve global food security goals - An overview

**DOI:** 10.1016/j.heliyon.2024.e25325

**Published:** 2024-02-02

**Authors:** Amir Afzal, Tariq Mukhtar

**Affiliations:** a Barani Agricultural Research Institute, Chakwal, Pakistan; b Department of Plant Pathology, Pir Mehr Ali Shah Arid Agriculture University, Rawalpindi, Pakistan

**Keywords:** Nematodes, Crop damage, Food security, Nematology research, Sustainable agriculture

## Abstract

Nematodes are soil-dwelling organisms that inflict substantial damage to crops, resulting in significant declines in agricultural productivity. Consequently, they are recognized as one of the primary contributors to global crop damage, with profound implications for food security. Nematology research assumes a pivotal role in tackling this issue and safeguarding food security. The pursuit of nematology research focused on mitigating nematode-induced crop damage and promoting sustainable agriculture represents a fundamental strategy for enhancing food security. Investment in nematology research is crucial to advance food security objectives by identifying and managing nematode species, developing novel technologies, comprehending nematode ecology, and strengthening the capabilities of researchers and farmers. This endeavor constitutes an indispensable step toward addressing one of the most pressing challenges in achieving global food security and promoting sustainable agricultural practices. Primarily, research endeavors facilitate the identification of nematode species responsible for crop damage, leading to the development of effective management strategies. These strategies encompass the utilization of resistant crop varieties, implementation of cultural practices, biological control, and chemical interventions. Secondly, research efforts contribute to the development of innovative technologies aimed at managing nematode populations, such as gene editing techniques that confer resistance to nematode infestations in crops. Additionally, the exploration of beneficial microbes, such as certain fungi and bacteria, as potential biocontrol agents against nematodes, holds promise. The study of nematode ecology represents a foundational research domain that fosters a deeper comprehension of nematode biology and ecological interactions. This knowledge is instrumental in devising precise and efficacious management strategies.

## 1 Introduction

As the global population swells towards 9–10 billion by 2050, ensuring food security while minimizing the adverse effects of food production on ecosystems has become a paramount challenge. One of the most pressing obstacles to this objective is the prevalence of plant diseases. These diseases can devastate crop yields, impair the quality and marketability of produce, and threaten the livelihoods of farmers and food producers. No major crop, whether it is a grain, fruit, vegetable, or cash crop, is immune from the impact of plant diseases, which can spread rapidly across regions and continents. This is particularly concerning in countries where smallholder farmers depend heavily on agriculture for their sustenance, as plant diseases can deepen poverty and exacerbate food insecurity by reducing crop yields, inflating input costs, and constraining market access for farmers [1,2].

Clearly, effective plant disease management strategies must be developed and implemented to promote food security and ensure sustainable intensification. A comprehensive, multifaceted approach is essential, encompassing prevention, surveillance, early detection, rapid response, and ongoing research and development [3,4]. By prioritizing and investing in plant disease management, we can establish a resilient, sustainable food system that can withstand the challenges of the future, thereby providing nourishment and security to all [1,2,5].

Fungi, bacteria, viruses, and nematodes are common plant pathogens that can cause significant damage to crops and affect food security [[6], [7], [8]]. Among the diverse plant pathogens, nematodes can cause severe damage to crops [9,10]. Plant parasitic nematodes are microscopic, worm-like organisms that live in the soil and feed on plant roots, leading to stunted growth, reduced yields, and in severe cases, plant death [11]. Nematodes are a major concern in agriculture due to their ability to cause significant damage to crops, resulting in reduced yields and economic losses for farmers [12]. They are particularly challenging to manage as they have a wide host range and can persist in the soil for many years, making it difficult to control their populations [13].

Research in nematology is critical to identifying and managing nematode species, developing new technologies, understanding nematode ecology, and building capacity in researchers and farmers [14,15]. Effective nematode management involves using a range of techniques, such as resistant crop varieties, cultural practices, biological control, and chemical control [[16], [17], [18]]. Gene editing and beneficial microbes are emerging technologies that hold promise in creating crops with resistance to nematode infestations [19]. Understanding nematode ecology is vital in developing precise and efficacious management strategies [20,21]. Studying nematode behavior and interactions with other organisms and environmental factors can provide valuable insights into nematode ecology. Investing in nematology research can promote sustainable agricultural practices and advance global food security [22].

## 2 The critical role of nematology research in addressing global food security challenges

The contribution of nematology research towards achieving the objective of global food security is significant. Nematodes are microscopic roundworms that reside in the soil and can inflict extensive crop damage, leading to a significant reduction in agricultural productivity. This poses a significant threat to global food security [23]. As such, research in nematology plays a crucial role in addressing this challenge and promoting sustainable agricultural practices [24].

Firstly, nematology research facilitates the identification of nematode species that cause crop damage and the development of effective management strategies to control their impact [25]. These strategies include the deployment of resistant crop varieties, cultural practices, biological control, and chemical control [26]. Such targeted and precise management strategies can help minimize the economic impact of nematode infestations on crop yields and promote sustainable agricultural practices. Furthermore, understanding the ecology of nematodes is a critical area of research that enables the development of more targeted and effective management strategies. Studying nematode ecology provides insights into the biology and ecological interactions of nematodes, which can be leveraged to develop precise and efficacious management strategies [27]. Such strategies not only help minimize the economic impact of nematode infestations on crop yields but also contribute to the long-term sustainability of agricultural practices.

Lastly, research in nematology plays a pivotal role in building the capacity of researchers and farmers in the field. This includes training in nematode identification, management strategies, and the use of new technologies. By building the capacity of researchers and farmers in nematology, sustainable agricultural practices can be promoted, and food security can be improved in the long run.

## 3 Economic significance of nematode diseases

Nematode infestations can cause extensive damage to crops by feeding on the roots, leading to stunted growth, wilting, and reduced yields [28]. This can lead to significant reductions in agricultural productivity, resulting in lower crop yields and financial losses for farmers. Moreover, nematode damage to crops can make them more susceptible to other diseases, leading to further losses [[29], [30], [31], [32], [33], [34], [35]]. Diseases caused by nematodes have significant economic implications for global agriculture. Plant parasitic nematodes cause significant crop losses worldwide, resulting in billions of dollars in annual losses. The estimated annual losses due to nematodes are around $157 billion globally [21,36,37]. This loss can be attributed to a range of factors, including crop damage, reduced crop yields, and increased production costs.

In addition to crop damage, nematode infestations can increase production costs by requiring the use of costly management strategies, such as chemical control [38]. This can add to the financial burden of farmers, particularly those in developing nations with limited resources. Nematode infestations can lead to food shortages, price fluctuations, and reduced export earnings, all of which have far-reaching socio-economic implications. In conclusion, the economic significance of diseases caused by nematodes is enormous, with billions of dollars lost annually due to crop damage and reduced yields. Investing in nematology research is essential in developing effective management strategies to mitigate these losses, promoting sustainable agriculture practices, and advancing global food security.

## 4 The importance of studying nematode ecology for effective pest management and sustainable agriculture

Studying nematode ecology is vital to understanding the behavior, distribution, and interactions of nematodes in the soil environment. Nematode ecology research involves investigating the biotic and abiotic factors that influence nematode populations, such as temperature, moisture, soil type, and plant species [27,[39], [40], [41]]. Such data are advantageous in developing effective nematode management strategies that reduce crop damage and bridge the gap between actual and potential yields.

By understanding how these factors impact nematode populations, researchers can develop targeted management strategies that exploit the nematodes’ weaknesses and reduce their impact on crops. Furthermore, nematode ecology research is essential in developing sustainable agricultural practices. By understanding the ecological interactions between nematodes, plants, and other soil organisms, researchers can develop integrated pest management strategies that minimize the use of chemical pesticides and promote natural pest control. Nematode ecology research also plays a crucial role in developing new technologies and tools for nematode management. For instance, understanding the genetic makeup of nematodes and their interactions with plant roots can inform the development of gene-editing tools that can confer natural resistance to nematodes in crops [9,42]. Therefore, studying nematode ecology is a fundamental research area that can inform the development of precise and efficient management strategies, promote sustainable agriculture practices, and lead to the development of novel technologies for nematode management.

## 5 Deploying resistant crop varieties: an effective and sustainable approach to nematode management

Developing and deploying resistant crop varieties are key management strategies against nematodes [[43], [44], [45], [46]]. This involves selecting and breeding crops with natural resistance to nematode infestations. Mitigating damage attributed to nematode infestation and leading to improved productivity can be achieved by selecting and breeding crops with natural resistance to nematode infestations in research institutes and agriculture universities. Resistant crop varieties work by limiting the ability of nematodes to feed on the plant roots, thereby reducing the severity of infestations [[47], [48], [49]]. Using resistant crop varieties is a sustainable and cost-effective way to manage nematode infestations, as it reduces the need for chemical control methods and helps maintain healthy soil ecosystems by reducing the impact of nematodes on the soil food web [50,51]. Resistant crop varieties can also help maintain soil health by reducing the need for soil fumigation, which can deplete soil nutrients and harm beneficial soil organisms [52,53]. The development of resistant crop varieties requires extensive research and breeding programs. Plant breeders use a variety of methods, including conventional breeding, genetic engineering, and marker-assisted selection to develop resistant varieties that can mitigate damage and improve productivity [54].

Researchers must identify natural resistance genes in crop plants and breed these genes into commercially viable varieties. The process can take several years and requires collaboration between researchers, breeders, and farmers. One of the challenges of deploying resistant crop varieties is the risk of nematode populations adapting to the resistance genes over time, which can lead to the emergence of new nematode strains that can overcome the resistance and cause damage to the crop [55]. To mitigate this risk, it is essential to deploy resistant crop varieties in conjunction with other management strategies, such as crop rotation and biological control. In short, deploying resistant crop varieties is an effective and sustainable approach to nematode management, but it requires significant investment in research and breeding programs and must be combined with other management strategies to ensure long-term effectiveness.

Currently, several methods are employed for breeding plants with nematode resistance, including conventional breeding, genetic engineering, and marker-assisted selection. Conventional breeding involves selecting and crossing parent plants with desirable traits, such as nematode resistance [56]. Genetic engineering is a newer technique that involves inserting genes from other organisms into crop plants to confer resistance to nematodes [57]. Marker-assisted selection is another newer method that uses molecular markers to identify plants with desired traits, such as resistance to nematodes [58,59]. This method can be faster and more precise than conventional breeding, but it is still in the early stages of development and use.

In general, these breeding methods are crucial in managing nematode populations in agriculture. By developing crop varieties with resistance to nematodes, farmers can reduce crop damage and improve productivity. However, it is important to use these methods in conjunction with other management strategies, such as crop rotation and cultural practices, to minimize environmental impact and reduce reliance on chemicals. The description of various breeding approaches is as follows.

### 5.1 Conventional breeding

Conventional breeding is a widely used process for developing crop varieties with desirable traits such as resistance to nematodes. The process involves identifying parent plants with natural resistance, crossbreeding to create hybrids, selecting for resistance, backcrossing with plants that possess desirable agronomic traits, and testing the resulting varieties under different conditions. This process can take several years to develop new resistant varieties, but the resulting varieties are often more effective in controlling nematode infestations than chemical control options and can provide long-term solutions for nematode management [60].

### 5.2 Genetic engineering

Genetic engineering presents another option for developing nematode-resistant crops. This method involves the insertion of genes from various sources, including naturally nematode-resistant bacteria and plants, into crop plants to confer resistance. Genetic engineering is often faster than conventional breeding and can lead to highly effective resistant varieties [42,57,61,62]. For instance, crops like cotton and corn have been genetically modified with a gene from *Bacillus thuringiensis* (Bt) bacterium to resist insect pests, which can indirectly reduce nematode populations. However, genetic engineering is still a controversial technology that raises concerns about its safety and environmental impacts [63].

While genetically modified crops have been proven safe for human consumption, there are worries about their potential impact on the environment and the risk of unintended consequences. For example, genetically modified crops might crossbreed with wild relatives, creating new invasive species. Additionally, the long-term effects of consuming genetically modified crops are not yet fully understood, and there is concern that they could have adverse effects on human health [[64], [65], [66]]. Therefore, the use of genetic engineering for developing nematode-resistant crops remains a subject of debate and ongoing research. Nevertheless, genetic engineering is a powerful tool for developing new crop varieties and has the potential to provide new solutions for managing nematode infestations in the future.

### 5.3 Marker assisted selection

Marker-assisted selection (MAS) is a plant breeding method that uses molecular markers to identify plants with desirable traits. In the case of nematode resistance, MAS can be particularly valuable, as nematodes are a significant challenge for many crops worldwide [67]. The use of MAS for nematode resistance has been successful in various crops, such as soybean, tomato, and banana [59,[68], [69], [70], [71], [72]]. For example, in soybean breeding, researchers used molecular markers to identify and select soybean plants with resistance to soybean cyst nematode (SCN). The selection of resistant plants using MAS resulted in improved resistance compared to conventional breeding methods [73]. Similarly, in tomato breeding, MAS was used to identify and select plants with resistance to root-knot nematode, resulting in the development of nematode-resistant tomato varieties [74]. In banana breeding, MAS has been used to develop varieties with resistance to nematodes such as *Radopholus similis* [75].

The use of MAS for nematode resistance offers several advantages over traditional breeding methods. It allows breeders to select for resistance at an early stage in the breeding process, saving time and resources. Additionally, MAS can select for multiple traits simultaneously, such as yield and nematode resistance, which can be challenging to achieve using conventional breeding methods [76,77]. Shortly, MAS has proved to be a valuable tool in developing nematode-resistant varieties of crops, and its application is likely to continue to increase in the future. By using molecular markers to select plants with desirable traits, breeders can develop varieties that are more productive, sustainable, and resistant to pests and diseases, contributing to global food security.

### 5.4 Using gene editing to develop nematode-resistant crops

Research in nematology has led to the development of innovative technologies for managing nematode populations, such as gene editing, which enables the creation of crops resistant to nematode infestations. Gene editing has emerged as a promising tool for developing nematode-resistant crops, and with advancements in technology, it is now possible to introduce targeted changes in the plant genome that confer resistance to nematodes. This process involves introducing specific changes in the DNA sequence of a plant, which alters the expression of genes involved in nematode resistance [25,42,78,79]. For example, researchers can introduce mutations that activate genes involved in the plant's defense against nematodes or modify existing genes to make them more effective against nematode infestations. Compared to traditional breeding methods, gene editing offers several advantages for developing nematode-resistant crops. It allows researchers to target specific genes involved in nematode resistance, making the process more precise and efficient. Additionally, gene editing can accelerate the development of new crop varieties, as traditional methods may take years to breed and test crops for resistance [42,75,[80], [81], [82], [83], [84]].

Despite its potential benefits, the use of gene editing in crop development remains controversial, with some critics raising concerns about the safety and ethics of genetically modified crops. Regulations governing the use of gene editing in agriculture also vary by country. Therefore, the technology must be used responsibly and in accordance with ethical and regulatory guidelines [[85], [86], [87], [88]]. In conclusion, gene editing offers a promising approach for developing nematode-resistant crops, but more research is needed to fully understand the long-term implications of this technology on agriculture and the environment.

## 6 Biological control of nematodes

Biological control is a sustainable and environmentally friendly approach to nematode management as it reduces the need for chemical control and promotes beneficial soil organisms. By developing and employing such technologies, researchers can help mitigate the negative impact of nematode infestations on crop yields, leading to improved food security. Beneficial microbes can be explored as a means of controlling nematodes. Biological control of nematodes involves the use of natural enemies, such as fungi, bacteria, and other organisms, to manage nematode populations [[89], [90], [91], [92], [93], [94], [95]].

One example of biological control is the use of nematophagous fungi, which are fungi that can infect and kill nematodes. These fungi can be applied to the soil in the form of spores, which then infect and kill the nematodes. Traditionally, nematophagous fungi have been categorized into four main groups based on their attacking mechanisms against nematodes [96]. Compared to bacteria and viruses, nematophagous fungi are particularly advantageous for the biological control of plant-parasitic nematodes [97,98]. Several significant fungal genera, such as *Arthrobotrys*, *Duddingtonia flagrans*, *Monacrosporium*, and *Dactylaria*, have been identified as effective against plant-parasitic nematodes [99]. The use of nematode-trapping fungi as biological control agents against phytonematodes has shown promising results [100]. Another example is the use of plant-parasitic nematodes, which can infect and kill other nematode species [28,38,89].

Biological control can also involve the use of beneficial microorganisms, such as rhizobacteria, which can colonize the plant roots and provide protection against nematode infestations. Rhizobacteria can promote plant growth, induce plant resistance, and compete with nematodes for resources, thereby reducing their populations [[101], [102], [103]].

Biological control methods involve utilizing living organisms, either in pure cultures or mixtures, to manage *Meloidogyne* spp [[104], [105], [106]]. Some biological products like those created by *Pasteuria* Inc. and Koppert Biological Systems against specific *Meloidogyne* spp. have exhibited significant efficacy in controlling these plant parasitic nematodes [[107], [108], [109]]. These products are generally derived from microorganisms such as *Pasteuria penetrans*, *P. hartismeri*, *Pochonia chlamydosporia*, *Bacillus firmus*, *Paecillomyces lilacinus*, and *Trichoderma* spp [43,89,94,[110], [111], [112], [114], [115], [116], [117], [118]]. These microorganisms function by attaching themselves to the nematode cuticle or by parasitizing female eggs, which ultimately results in the nematode's demise [89,114,115,119]. Additionally, some studies have demonstrated an alternative biological strategy in which endophytes like *Fusarium oxysporum* (FO162) can activate systemic resistance against *Meloidogyne* spp. in certain crops such as tomatoes [120,121]. Root colonization by FO162 triggers the accumulation of root exudates in tomato roots, which have a repellent impact on *M. incognita* [122].

One of the advantages of biological control is that it can be used in conjunction with other management strategies, such as crop rotation and resistant crop varieties [[123], [124], [125], [126]]. This can help to create a more holistic and effective approach to nematode management. However, the success of biological control depends on several factors, including the selection and deployment of appropriate biological control agents, environmental conditions, and the overall health of the soil. Additionally, the effectiveness of biological control can be limited by the natural variability of nematode populations and their ability to adapt to new environments [[127], [128], [129], [130]]. In conclusion, biological control is an effective and sustainable approach to nematode management that can reduce the need for chemical control and promote beneficial soil organisms. However, its effectiveness depends on several factors and must be used in conjunction with other management strategies to ensure long-term success**.**

## 7 Effective cultural practices for nematode management

Cultural practices can be an effective means of managing nematode populations, reducing crop damage, and promoting sustainable agriculture practices. Cultural practices refer to a range of agricultural practices that can be used to manage nematode populations. These practices, when used in combination with other management strategies such as biological control and chemical control, can help mitigate the economic impact of nematode diseases on global agriculture. These practices involve altering the cropping system, soil management, and other agricultural practices to reduce nematode populations and their impact on crops [[131], [132], [133]].

### 7.1 Crop rotation as a key cultural practice

One of the most effective cultural practices for nematode management is crop rotation. This involves alternating the planting of crops that are susceptible to nematode infestations with crops that are resistant to nematodes. By rotating crops, farmers can break the nematode life cycle, reducing their populations and preventing them from building up in the soil. It is based on the principle that different crops have different susceptibility to nematodes, and that by rotating crops, the nematodes are deprived of their host plants, and their populations decline [[134], [135], [136]]. In addition to reducing nematode populations, crop rotation can also improve soil health, fertility and structure, leading to improved crop growth and yields [[137], [138], [139], [140], [141], [142]]. The economic viability of rotation crops is an important consideration for farmers [[143], [144], [145]]. In addition to providing resistance to nematode infestations, rotation crops should also be profitable and contribute to the overall income of the farm. The yield increases in a subsequent crop after rotation should be sufficient to justify the use of land for the rotation crop. This is why it is important to carefully select rotation crops that are both resistant to nematodes and economically viable [146,147].

The manifestation and population levels of plant-parasitic nematodes fluctuate depending on the crop grown. This means that cultivating a rotation crop that is resistant to one nematode species may unconsciously endorse another species, which could become the dominant pathogen. Hence, it is decisive to conduct regular sampling to recognize the dynamics of different nematode species in a field [18]. Furthermore, effective weed management is crucial to prevent nematode increases on weed host plants [148,149].

In some cases, it may be necessary to use a combination of cultural practices, such as crop rotation and cover cropping, to effectively manage nematodes and maintain the economic viability of the farm. Crop rotation should be combined with other nematode management practices such as the use of resistant cultivars, sanitation and soil solarization for best results [137,[150], [151], [152], [153]].

### 7.2 Cover crops for nematode management and soil health

Another cultural practice is the use of cover crops, which are planted between cash crops to improve soil health and reduce nematode populations [137,[154], [155], [156], [157]]. Cover crops provide a habitat for beneficial microbes that can help control nematodes, and they also improve soil structure, which can reduce the impact of nematode damage on crops [38,137,[158], [159], [160]]. Studies have shown that population densities of *Rotylenchulus reniformis*, a nematode species known to cause significant damage to cotton crops, can be reduced under *R. reniformis*-resistant soybean to such levels that subsequent susceptible cotton crops are protected from extensive nematode damage [146,161].

### 7.3 Deep tillage in nematode management

Soil management practices such as deep tillage, which involves turning over the soil to expose nematodes to the elements, can also help reduce nematode populations. Deep tillage is a soil management practice that can help reduce nematode populations by exposing them to the elements. By disturbing the soil, nematodes are brought to the surface where they are exposed to conditions such as sunlight and drying that can be harmful to them [131,153,162]. However, this practice can be detrimental to soil health and is not recommended in all situations. Deep tillage can lead to soil erosion, loss of organic matter, and disruption of soil structure, which can ultimately harm soil health and reduce crop yields. Therefore, it is important to carefully consider the potential benefits and drawbacks of deep tillage before implementing it as a nematode management strategy. In some cases, reduced tillage or no-till practices may be more appropriate and effective for maintaining soil health while still managing nematode populations [131,156,[163], [164], [165], [166]].

### 7.4 Organic amendments for nematode management

Other cultural practices include the use of organic amendments such as compost and manure, which can improve soil health and stimulate the growth of beneficial microbes [[167], [168], [169]]. Organic amendments can enhance soil fertility and biological activity, leading to a reduction in nematode populations [43,[170], [171], [172]]. For instance, compost has been found to increase populations of soil-dwelling predatory mites, which can feed on and reduce nematode populations. Similarly, the application of manure has been shown to suppress nematode populations by improving soil microbial activity and nutrient availability [173].

### 7.5 Usage of trap crops for nematode management

Trap crops can serve as a sacrificial crop, luring nematodes away from the main cash crop and reducing their population. These crops are usually highly susceptible to nematodes and are planted as a border around the main crop. The nematodes are attracted to the trap crop and will reproduce on it, but the crop is later removed, reducing the nematode population in the soil before the main crop is planted. This practice can be particularly effective when used in combination with crop rotation and other integrated pest management strategies. By employing a variety of cultural practices, farmers can reduce their reliance on chemical nematicides and promote long-term sustainability of their soil and crops [174].

## 8 Chemotherapy for management of nematodes

Chemical control is a commonly used management strategy against nematodes in agriculture due to its immediate effect on nematode populations. Nematicides are chemical compounds designed to kill nematodes and are often used in crops where other management strategies may not be feasible or effective enough to control nematode infestations. They can be applied to the soil or plant roots to reduce nematode populations and limit crop damage. Chemical control is often used in combination with other management strategies, such as crop rotation and resistant crop varieties, to maximize efficacy and minimize environmental impact [[175], [176], [177], [178]].

Nematicides are usually classified into two types based on their mode of action: contact nematicides and systemic nematicides. Contact nematicides work by coming into direct contact with the nematodes in the soil, killing them on contact. They are typically less toxic than systemic nematicides and have a shorter residual effect. Systemic nematicides, on the other hand, are absorbed by the plant roots and translocated throughout the plant. They are highly effective but can have a longer residual effect and may be more toxic [[175], [176], [177],^179^].

Overall, chemical control can be an effective management strategy against nematodes in agriculture, but it should be used with caution and in conjunction with other strategies to minimize environmental impact and reduce the risk of resistance development. Research in alternative, more sustainable control methods, such as biological control, is crucial for advancing the field of nematology and promoting sustainable agriculture practices.

## 9 Conclusion

Food insecurity remains a pressing global issue, particularly in developing countries in Africa, Asia, and Latin America, where plant parasitic nematodes pose a significant threat to agriculture and global food security. With their potential to cause substantial damage to crops and result in reduced yields and economic losses for farmers, it is essential to characterize these harmful nematodes to develop effective and sustainable control strategies. Nematologists play a critical role in advancing our understanding of nematodes and developing new technologies and methods for managing infestations, enhancing ecological knowledge, and building research and farmer capacity.

By investing in nematology research, we can make significant strides toward ensuring food security for millions of people in affected countries. While assessing the impact of nematode infestations on crop yields can be challenging, researchers can increase our knowledge of nematodes and develop innovative strategies for their control. Nematologists are well-positioned to meet this challenge, and the future of nematology lies in advancing research and development to promote food security and combat plant parasitic nematodes.

In conclusion, investing in nematology research is crucial for achieving the goal of global food security. By identifying and managing nematode species, developing new technologies, understanding nematode ecology, and building the capacity of researchers and farmers, we can mitigate the negative impact of nematode infestations on crop yields and promote sustainable agricultural practices. As such, nematology is an essential field of research that has far-reaching implications for global food security and should be a priority for continued investment and advancement.

## Funding statement

There was no funding.

## Data availability statement

The data associated with the study has not been deposited into a publicly available repository.

Data included in article/supp. material/referenced in article.

## Human and animal rights

This article does not contain any studies with animals performed by any of the authors.

## CRediT authorship contribution statement

**Amir Afzal:** Writing – review & editing, Writing – original draft, Data curation, Conceptualization. **Tariq Mukhtar:** Writing – review & editing, Validation, Supervision, Data curation, Conceptualization.

## Declaration of competing interest

The authors declare that they have no conflict of interest.
